# Six Imprisoned Health-Care Workers in Libya Are Pawns in a Far Larger Strategic Game: The repercussions are enormous

**DOI:** 10.1371/journal.pmed.0030514

**Published:** 2006-11-21

**Authors:** Laurie Garrett

## Abstract

It is critical that the scientific community recognize what is at stake in the case of the Benghazi Six, says Laurie Garrett of the Council on Foreign Relations.

The scientific community, AIDS activists, and the Libyan government would do well to recognize that the political and diplomatic import of the case of the Benghazi Six involves a great deal more than the lives of five Bulgarian nurses and a Palestinian physician. At stake are some of the most profound political issues of our time: terrorism, nuclear proliferation, the freedom of movement of health-care workers and scientists, and the Biological Weapons Convention. Although human rights advocates rightly decry the physical torture these individuals have been subjected to, and their death sentences, it is critical to recognize that the unfortunate Benghazi Six—Bulgarian nurses Snezhana Dimitrova, Nasya Nenova, Valentina Siropulo, Valya Chervenyashka, and Kristiyana Valtcheva, and Palestinian physician Ahmed Ashraf Al Hadjudi—are pawns in a far larger game.

## The Geopolitical Background

In 2003 Colonel Muammar Gaddafi, the longtime leader of Libya, initiated discussions through American and European diplomatic channels signaling interest in placing Libya within the larger world community. Isolated under the labels of “rogue state” and “supporter of terrorism,” Libya was constrained by United Nations sanctions that, among other things, limited that country’s ability to pump and sell its vast oil reserves or to purchase the vital electronics and equipment needed to modernize its oil fields. Breaking those constraints meant renouncing all ties to terrorism and admitting responsibility for the 1988 explosion of Pan Am Flight 103, the jet that crashed into Lockerbie after a bomb placed in the jet’s cargo hold by Libyan operatives exploded, claiming the lives of 270 people. Gaddafi, after initially denying any Libyan responsibility for the international crime, in 1999 handed over one of his intelligence officers for trial in Scotland, where he remains in prison today.

Pained by the sanctions, in 2003 Libya formally acknowledged to the United Nations Security Council responsibility for the Lockerbie bombing, and began negotiations with representatives of the families of the Flight 103 victims. Libya ultimately paid the families US$2.16 billion in 2005; another US$540 million in promised payment was withdrawn by Libya because the United States Bush Administration maintained the country on its terrorism watch list.

At stake are some of the most profound political issues of our time.

In addition, the Gaddafi government has quietly admitted to working with Pakistani nuclear weapons scientist Dr. Abdul Qadeer (“A. Q.”) Khan. Libya has not only abandoned its nuclear weapons dreams, but has cooperated in international investigations of A. Q. Khan’s dangerous spread of nuclear weapons–related knowledge and equipment to a laundry list of states.

Libya is now on a path to joining the world as a global citizen. But the process is far from complete. On May 16, 2006 US Secretary of State Condoleeza Rice announced that the US and Libya were initiating normalization of relations. But America has not yet positioned an Ambassador in Tripoli, as normalization is a multistaged process that could drag out for many years if either of the two countries is dissatisfied with the proceedings. Many players are observing the process closely, including the European Union and the UN Security Council. If any major player believes Libya is reneging on agreements or acting in bad faith, the normalization process could be imperiled.

The stakes are very high for Libya, as the nation is desperate to play a dominant role in the global petroleum market, to modernize, and to become a technological leader in the Middle East. At a time when the Gulf States are building large universities modeled after MIT and Harvard, Libya has a per capita gross national income equivalent to US$5,500, is unwilling to provide adult literacy data to the UN, and has a population dominated by children—30% of Libyans are under 14 years of age [1].

It is in Libya’s urgent interests to acquire an image of openness to scientific exchange and expertise. But Libya must demonstrate that, first, it will not use such scientific openness to acquire the capacity to produce weapons of mass destruction, and second, that it will respect the human rights of foreign professionals who work on Libyan soil.

## The Benghazi Six

Imprisoned since 1999, the five Bulgarian nurses and Palestinian physicians “confessed” to the crime of working with the CIA to deliberately infect 426 Libyan children with HIV. Their confessions were extracted after extensive torture, some of which was eye-witnessed by a Bulgarian engineer who was jailed simultaneously for 174 days on unrelated charges. On May 16, 2006 the engineer, Smilian Tachev, held a press conference in Sofia, Bulgaria, revealing the conditions to which the Benghazi Six were subjected:

“The nurses were beaten with many-stranded wire, for a long time and painfully,” Tachev said. “Then they were made to run, crawl, stand on one leg with their hands stretched up. When they collapsed totally, they were dragged somewhere and brought back in a helpless state.” Tachev added that he witnessed the use of probes to force unidentified objects down the women’s throats, electrocution, and dogs loosed on the screaming victims [2].

For seven years the nurses and doctor have been imprisoned, facing a sequence of Libyan judicial proceedings, and in 2004 were sentenced to death by firing squad. By all accounts their lives have taken this hideous turn for arbitrary reasons. When it was revealed in 1998 that 426 children that had been hospitalized in a facility in which the Benghazi Six worked were now HIV positive, the Gaddafi government rounded up every foreign-born physician, nurse, and technician employed in the facility. Though local medical personnel decried the unsanitary conditions of the hospital, and blamed reused syringes for the spread of HIV among pediatric patients, the Libyan government charged these six with a crime and released the other foreigners [3]. In early 2006 Gaddafi added another name to the list of alleged criminals—Switzerland’s prominent AIDS researcher Luc Perrin, who had examined some of the infected children and studied their blood samples in his Geneva University Hospital laboratory, years after the Benghazi Six were arrested [4].

Emotions have reached fever pitch among the families of the HIV-infected children. The families have held demonstrations calling for the health-care workers’ executions, burning American and Bulgarian flags. And they have insisted that Bulgaria and the US must make payments to the children’ families that are equal to the amounts Libya paid the Lockerbie victims’ survivors. Bulgaria and the US refuse.

## What is At Stake for the Health and Scientific Communities

Meanwhile, the stakes are high for scientists and health-care workers, generally. The world is shy 4.3 million health-care workers, with the greatest deficits being felt in poor countries hard-hit by HIV, tuberculosis, and malaria [5]. If there is any hope of conquering the AIDS pandemic, physicians, nurses, technicians, and scientists must be free to work in countries other than their home of citizenship. In recent years, however, we have witnessed numerous incidents in which governments or religious leaders targeted foreign health professionals as part of larger political schemes: Nigerian imams, for example, claimed American-made polio vaccines contained HIV, spawning a global resurgence of polio [6]. Freedom for the Benghazi Six would move the world towards restoring principles of free movement for legitimate health-care workers and scientists.

Indeed, the HIV-positive children of Libya deserve access to the same quality of medical care as their pediatric counterparts in Europe and North America enjoy. The best way for them to obtain years of quality life is through guarantees that doctors, nurses, scientists, and pharmacists, expert in HIV/AIDS treatment, have safe access to their country and its hospitals.

Libya is fortunate that Bulgaria, then a young post-communist state, did not insist in 1999 that charges be filed with the Biological Weapons Convention. Bulgaria should have done so. After all, Gaddafi claimed that Bulgaria and the US CIA colluded in a fiendish plot to deliberately release a microorganism into the Libyan population. Had the claim been processed as a formal charge, weapons inspectors would have had formal access to blood samples, hospital records, and other vital information that would undoubtedly have cleared the Benghazi Six. Moreover, a signal would have been sent to the world regarding claims of bioterrorism and the burden of their proof [7]. In the event, Libya’s failure to invoke the Biological Weapons Convention to fully investigate the criminal allegations undermines the credibility of Gaddafi’s charges and the convictions of these health-care workers.

It is critical that the scientific community recognize what is at stake in this case: It is your freedom of movement and work; it is the strength and validity of the Biological Weapons Convention; it is Libya’s laudable willingness to remove itself from the list of nations that support terrorism and seek nuclear weapons capability. And it is freedom for six unjustly treated colleagues.
